# Microstructural Evaluation and Linkage to the Engineering Properties of Metal-Ion-Contaminated Clay

**DOI:** 10.3390/ma17215320

**Published:** 2024-10-31

**Authors:** Yikun Chen, Ya Chu, Chao Yan, Wei Duan, Aimin Han

**Affiliations:** 1 College of Transportation Engineering, Nanjing Tech University, Nanjing 211816, China; 2 Anhui Institute of Expansive Soil Mechanics and Engineering, Hefei 236025, China; 3 College of Civil Engineering, Taiyuan University of Technology, Taiyuan 030024, China

**Keywords:** alkali metal ions, heavy metal ions, contaminated clay, microstructural, macro-mechanical, pore structure evaluation parameters, quantitative analysis

## Abstract

The rapid progress of urbanization and industrialization has led to the accumulation of large amounts of metal ions in the environment. These metal ions are adsorbed onto the negatively charged surfaces of clay particles, altering the total surface charge, double-layer thickness, and chemical bonds between the particles, which in turn affects the interactions between them. This causes changes in the microstructure, such as particle rearrangement and pore morphology adjustments, ultimately altering the mechanical behavior of the soil and reducing its stability. This study explores the effects of four common metal ions, including monovalent alkali metal ions (Na^+^, K^+^) and divalent heavy metal ions (Pb^2+^, Zn^2+^), with a focus on how ion valence and concentration impact the soil’s microstructure and mechanical properties. Microstructural tests show that metal ion incorporation reduces particle size, increases clay content, and transforms the structure from layered to honeycomb-like. Small pores decrease while large pores dominate, reducing the specific surface area and pore volume, while the average pore size increases. Although cation exchange capacity decreases, cation adsorption density per unit surface area increases. Monovalent ions primarily disperse the soil structure, while divalent ions induce coagulation. Macro-mechanical tests reveal that metal ion contamination reduces porosity under loading, with compressibility rises as the ion concentration increases. Soils contaminated with alkali metal ions shows higher compression coefficients at all loads, while heavy metal ions cause higher compression under lower loads. Shear strength, the internal friction angle, and cohesion in metal-ion-contaminated clay decrease compared to uncontaminated field-state clay, with greater declines at higher ion concentrations. The Micropore Morphology Index and hydro-pore structural parameter effectively characterize both micro- and macrostructural properties, establishing a quantitative relationship between HPSP and the engineering properties of metal-ion-contaminated clay.

## 1. Introduction

With the rapid growth of the global economy, issues such as excessive industrial waste discharge, overuse of pesticides and fertilizers, and chaotic landfill practices have intensified. Contamination of inorganic metal-ion-contaminated clay (MICC) has emerged as a global challenge, with complex origins, far-reaching impacts, and significant remediation difficulties. International scholars are conducting research from diverse perspectives—including materials [1,2], microbiology [3,4], botany [5], and electrochemistry [6]—aiming to remove metal ions from clay and restore its natural condition.

It is important to recognize that the intrusion of metal ions not only threatens ecological safety but also significantly weakens the engineering properties of soil [7,8]. This can lead to geotechnical problems, such as uneven foundation settlement and landslides, posing serious risks to infrastructure safety and social stability. At the microstructural level, different metal ions have varying effects on soil particles. The addition of Na^+^ causes the pores in the soil to develop from linear to planar forms [9]. The introduction of Zn^2+^ reduces the thickness of the bound water layer on the particle surfaces [10], leading to the gradual disappearance of flocculent colloids between particles, and the soil particle structure begins to exhibit a honeycomb-like characteristic [11]. Similarly, the infiltration of Pb^2+^ weakens the soil structure, causing particle connections to loosen [10]. At low concentrations, Cu^2+^ dissolves the cementing materials between particles, thinning the bound water layer and causing disordered particle aggregation. However, as the concentration of Cu^2+^ increases, the bound water layer thickens again, and the aggregated particles transform into a sheet-like structure [12]. The addition of As^3+^ increases the absolute value of the Zeta potential of the soil, resulting in an increase in the number of small pores. The fractal dimension of the pores first decreases and then increases with an increasing arsenic concentration. In contrast, Cd^2+^ reduces the absolute value of the Zeta potential, increases the number of large pores, and significantly lowers the fractal dimension of the pore structure [13].

In terms of engineering properties, metal ions generally weaken the stability of the soil. Na^+^ increases the optimal water content of the soil while reducing the internal friction angle and cohesion [9]. Zn^2+^ significantly decreases the unconfined compressive strength and shear strength of the soil [10,11,14], while increasing the internal friction angle and decreasing cohesion, which leads to higher compressibility [15] and an increase in shrinkage rate [16]. Pb^2+^ contamination reduces the clay content of the soil, resulting in an increase in maximum dry density, optimal water content, void ratio, and compression coefficient, while the expansion rate, shear strength, and unconfined compressive strength decrease significantly [14,17]. Even when Pb^2+^-contaminated soil is treated with Microbially Induced Calcium Carbonate Precipitation (MICP) solidification technology, although there is some improvement in strength, the soil remains constrained by lead contamination [18]. Particularly after freeze–thaw cycles, the number of micropores increases, the strength further declines, and leachate concentrations rise [19]. As Cu^2+^ concentration increases, the soil’s compression coefficient and total compression deformation initially decrease and then increase [12].

Current research on MICC primarily focuses on heavy metal ions and their concentration variations. However, there is a lack of comprehensive studies on the differences between metal ions with different valences and the quantitative relationship between microstructural changes and macro-mechanical properties. Therefore, there need for a deeper exploration of the engineering characteristics of MICC and its intrinsic evolution mechanisms has become a pressing issue, critical to ensuring engineering safety. Accordingly, this study systematically investigated the microstructural changes and their impact on the engineering properties of MICC, with a focus on the effects of different ion valences and concentrations. A microstructural characterization method for MICC’s engineering properties is proposed, establishing a quantitative relationship between microstructural characteristics and macro-mechanical properties. This research holds significant academic and engineering value, providing robust theoretical support for engineering safety, environmental protection strategies, and ecological restoration technologies.

## 2. Materials and Methods

### 2.1. Clay Properties and Selection of Metal Ions

**Figure 1:**
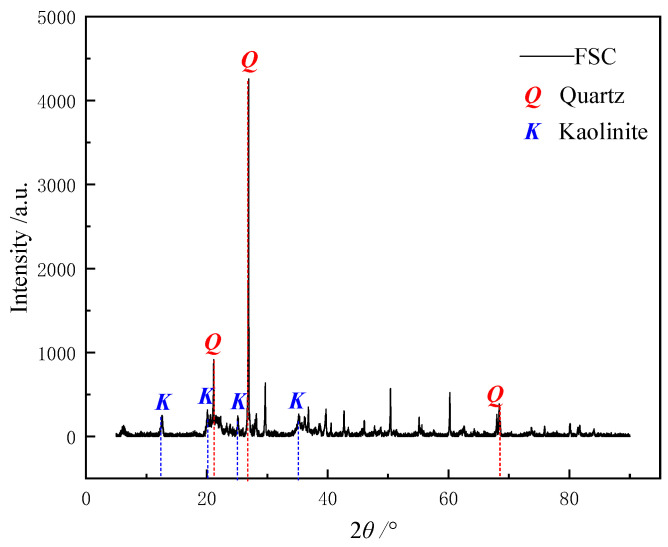
X-ray diffraction image of FSC.

The clay used in this experiment was collected from Nanjing, China. Nanjing Field-State Clay, a floodplain silty clay, has been subjected to prolonged exposure to river erosion and sedimentation, resulting in a relatively homogeneous composition with small and narrowly distributed particle sizes. X-ray diffraction (XRD) analysis was performed on the mineral composition of the FSC using an X-TRA model X-ray diffractometer from Thermo Fisher Scientific, Waltham, MA, United States. The diffraction patterns were recorded with a scanning range of 5° to 90° 2θ, at a scanning speed of 2° per minute. The X-ray source operated at a voltage of 40 kV and a current of 40 mA. Structural information about the mineral components was obtained by comparing characteristic peaks through Jade 5.0 analysis software. X-ray diffraction (Figure 1) analysis showed that the mineral composition mainly consists of quartz and kaolinite. In a typical cohesive soil, kaolinite particles usually carry a negative surface charge, making them highly susceptible to the influence of metal ions. After passing through a 0.5 mm sieve, all particles were smaller than 100 μm, primarily consisting of clay or silt-sized particles. The plastic limit of Nanjing clay was 28, the liquid limit was 42, and the plasticity index was 18, indicating relatively lower plasticity compared to common clays. Overall, it exhibited a uniform mineral composition, narrowly distributed particle sizes, and a stable structure.

Sodium ions (Na^+^) and potassium ions (K^+^) were selected as representative monovalent alkali metal ions, which are common in soils, particularly in agricultural fields and saline alkali environments due to fertilizer use. Lead ions (Pb^2+^) and zinc ions (Zn^2+^) were chosen as divalent heavy metal ions because of their significant environmental pollution risks. Pb^2+^ is commonly found in emissions from transportation, wastewater, and mining, while Zn^2+^ is a byproduct of many industrial processes.

Nanjing, as a representative industrialized city in China, is surrounded by a concentration of manufacturing and heavy industry. These four metal ions are also the primary pollutants in and around Nanjing. Many cities around the world share a similar industrial profile to Nanjing. Therefore, sodium, potassium, lead, and zinc ions are not only major pollutants in Nanjing and its surrounding areas but are also common contaminants in many industrialized cities globally, making them key targets for environmental remediation and soil management.

In summary, this study selected typical clay from Nanjing, China, as the research objects. Monovalent sodium and potassium ions, as well as divalent lead and zinc ions, were introduced as contaminating agents. Tests were conducted to analyze the microstructural characteristics and physical–mechanical properties of the clay.

### 2.2. Preparation of Metal-Ion-Contaminated Clay (MICC)

The experimental MICC samples were collected from floodplain silty clay in Nanjing City, Jiangsu Province, China. After removing impurities, drying, and grinding, particles smaller than 0.5 mm were selected for the preparation of different MICC types. Four metal ions were chosen for the experiment: Na^+^, K^+^, Pb^2+^, and Zn^2+^. Na^+^ and K^+^ are monovalent alkali metal ions, while Pb^2+^ and Zn^2+^ are divalent heavy metal ions. According to the “Soil Environmental Quality Risk Control Standards for Soil Contamination of Development Land in China” (GB 36600-2018) [20], three concentrations were set at 500 mg/kg, 5000 mg/kg, and 25,000 mg/kg (mass of target ions/mass of dry clay), resulting in a total of 12 MICC samples. Considering factors such as good water solubility, chemical stability, and the need to avoid reactions with other components in the soil, sodium chloride, potassium chloride, lead nitrate, and zinc nitrate were ultimately selected as the contaminating compounds.

According to the experimental requirements, a sufficient quantity of MICC was prepared. The respective metal compounds were accurately weighed based on the relative atomic masses of the ions and dissolved completely in deionized water. The solution volume was carefully controlled to ensure the MICC achieved a plastic consistency. The metal ion solution was gradually added to the MICC, thoroughly mixed, and cured for 15 days to ensure complete adsorption of the metal ions onto the particle surfaces. After curing, the MICC was dried, ground, and screened again, with particles smaller than 0.5 mm selected for subsequent studies.

**Figure 2:**
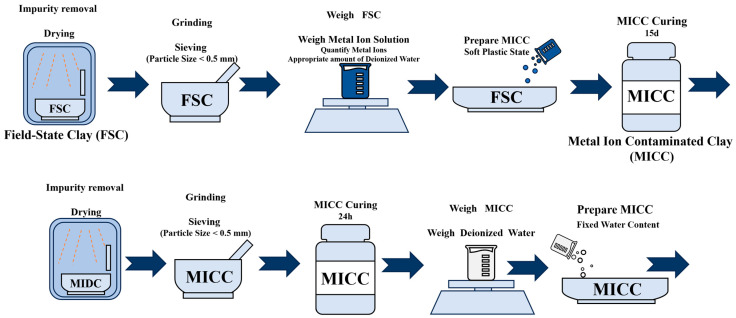

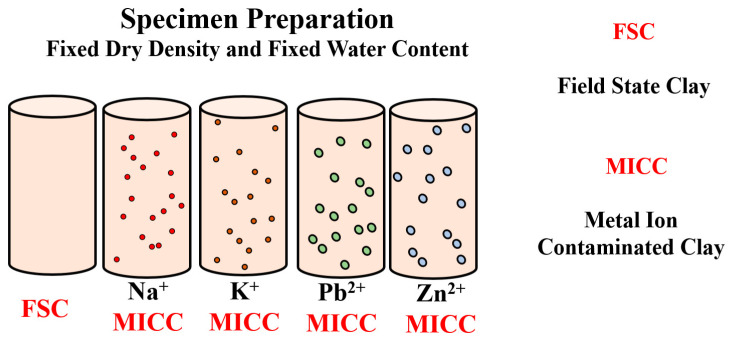
Schematic diagram of MICC preparation process.

**Figure 3:**
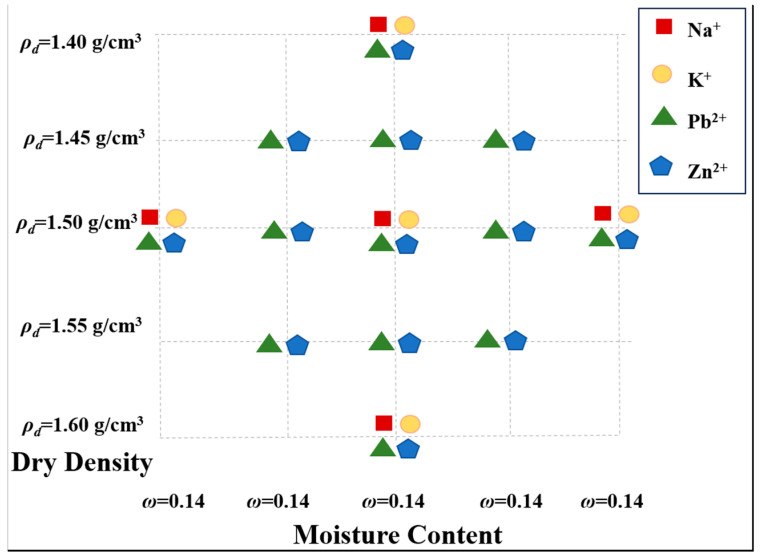
MICC moisture content—dry density configuration chart.

To explore the effects of moisture content and structural properties on the mechanical characteristics of MICC, five samples with water contents of 14%, 18%, 22%, 26%, and 30% were prepared using deionized water, based on the plastic limit of uncontaminated MICC. These samples were cured for 24 h. Additionally, five MICC samples with dry densities of 1.40 g/cm^3^, 1.45 g/cm^3^, 1.50 g/cm^3^, 1.55 g/cm^3^, and 1.60 g/cm^3^ were prepared to ensure that the pore saturation of the samples showed an increasing trend. The overall experimental configurations are shown in Table 1, with the preparation process illustrated in Figure 2 and the dry densities and moisture contents shown in Figure 3.

### 2.3. Experimental Procedures

The prepared metal-ion-contaminated clay (MICC) samples underwent tests to evaluate their microstructure and engineering properties, providing a comprehensive assessment of the impact of metal ions on the characteristics of MICC. In the microstructure tests, scanning electron microscopy (SEM) was employed to examine the micro-morphology, analyzing surface features, agglomeration phenomena, and pore structures of the particles after doping. Particle size analysis determined the distribution of particle sizes before and after doping, evaluating the influence of metal ions on the dispersion and agglomeration of MICC particles. Nitrogen adsorption methods were used to measure specific surface area and mesopore parameters, revealing changes in surface area, pore volume, and average pore size due to metal ion modification. Cation exchange capacity (CEC) tests were conducted to quantify the cation adsorption capacity on the MICC particle surfaces, assessing the effect of metal ion doping on the charge characteristics of MICC.

In the engineering property tests, standard consolidation and direct shear tests were performed. The consolidation tests analyzed changes in void ratio and compressibility by measuring compression under different load conditions. The direct shear tests determined the shear strength, internal friction angle, and cohesion of MICC after doping, highlighting the effects of metal ions on its shear behavior. These experiments systematically explored the mechanisms by which metal ion doping affects the microstructure and mechanical properties of MICC.

#### 2.3.1. Microstructure Testing

The particle distribution of the MICC samples was analyzed using a Malvern Mastersizer 2000 laser particle size analyzer, manufactured by Malvern Panalytical, Worcestershire, United Kingdom, conducting dry particle size analysis. Specific surface area and porosity parameters were measured using the Autosorb NOVA 2200e, manufactured by Quantachrome, Boynton Beach, FL, United States. Nitrogen adsorption/desorption experiments were conducted at 1 atm (101,325 Pa) and 77.3 K (−196.15 °C) to obtain parameters such as specific surface area, pore volume, and average pore diameter. Scanning electron microscopy (SEM) testing was performed using the Hitachi Regulus 8100 model, produced by Hitachi High-Tech Corporation, Tokyo, Japan. To enhance surface conductivity and improve imaging quality, gold ion sputtering was applied during sample preparation. The SEM was operated at an accelerating voltage of 15 kV, with magnifications set between 3000× and 4500× to clearly observe the microstructural characteristics of the particles. Cation exchange capacity (CEC) was determined using the Soil quality—Determination of cation exchange capacity (CEC)—Hexammine cobalt trichloride solution–Spectrophotometric method for soil CEC testing (HJ 889-2017) [21].

#### 2.3.2. Engineering Property Testing

Standard consolidation tests were conducted to determine the compression characteristics of the metal-ion-contaminated clay (MICC) under confined conditions. Standard Test Methods for One-Dimensional Consolidation Properties of Soils Using Incremental Loading (ASTM D2435-2020) [22] and the Standard for Soil Testing Method (GB/T 50123-2019) [23] were applied utilizing the GZQ-1A fully automatic air pressure consolidation apparatus, developed by the Nanjing Soil Research Institute. Test samples had a diameter of 61.8 mm and a height of 20 mm, with consolidation pressure levels set sequentially at 50 kPa, 100 kPa, 200 kPa, 400 kPa, and 800 kPa. The deformation of the samples at each pressure level was recorded until stabilization, with a consolidation rate of less than 0.01 mm/h indicating the completion of compression at that pressure level.

To further investigate the shear strength of the MICC, standard direct shear tests were performed. These tests followed the Standard Test Method for Direct Shear Test of Soils Under Consolidated Drained Conditions (ASTM/D3080M-23) [24] and the Standard for Soil Testing Methods (GB/T 50123-2019) [23], using the ZJ strain-controlled direct shear apparatus (four-unit), developed by the Nanjing Soil Research Institute. The test samples were the same size as those used in the consolidation tests (61.8 mm in diameter and 20 mm in height). Vertical pressure levels were set at 50 kPa, 100 kPa, 200 kPa, and 400 kPa, with a shear rate of 0.8 mm/min. The peak shear force was recorded at each pressure level, or the shear force when shear displacement reached 4 mm. Using the Mohr–Coulomb strength theory, the internal friction angle and cohesion of the samples were calculated.

## 3. Results and Analysis

This chapter presents the results of tests on the microstructure and macro-mechanical properties of MICC under varying concentrations. The microstructural characterization focused on changes in particle morphology, particle size distribution, pore structure, and cation adsorption on particle surfaces. Macro-mechanical tests evaluated key performance parameters such as shear strength and compressibility of MICC. The results demonstrate a strong correlation between microstructural and macro-mechanical properties, offering new insights into the mechanisms by which metal ions influence MICC.

### 3.1. Microstructure Test Results

This study conducted a detailed analysis of the MICC microstructure under varying conditions. Various microstructural testing methods, including laser particle size analysis, SEM, specific surface area and mesopore analysis, and cation exchange capacity (CEC) testing, revealed the arrangement of particles, pore structure, and ion adsorption density at different concentrations of metal ion doping. Microstructural changes directly reflect the effects of metal ions on particle bonding strength and pore structure, providing a theoretical basis for subsequent macro-mechanical performance analysis.

#### 3.1.1. Particle Size Distribution Curve

**Figure 4:**
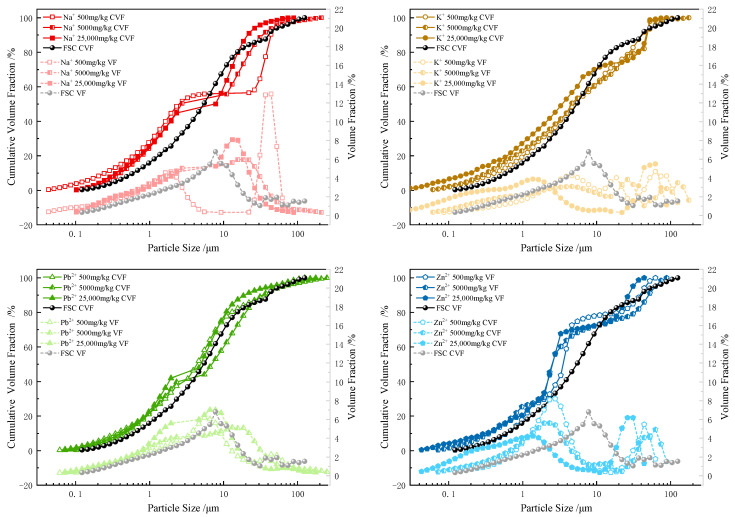
Cumulative volume fraction and volume fraction of particle size distribution for FSC and MICC (Na^+^, K^+^, Pb^2+^, Zn^2+^).

As shown in Figure 4, the incorporation of metal ions significantly altered the original particle size distribution characteristics of the MICC, resulting in distinct segments in the cumulative volume fraction curve and an increase in the number of peaks in the volume fraction curve. Specifically, the cumulative volume fraction curve of the FSC is smooth, while the volume fraction curve shows a peak at 8 μm. However, after the addition of metal ions, the cumulative volume fraction curves for sodium, lead, and zinc ion-contaminated MICC exhibit significant segmentation at 2 μm. Additionally, the volume fraction curves for sodium, potassium, and zinc ion-contaminated MICC increase in peak count to two, roughly distributed in the ranges of 1–2 μm and 12–14 μm.

FSC contains various particle types, such as clay particles, sand grains, and rock fragments, forming a relatively stable particle size distribution during consolidation or deposition. When metal ions are introduced, the fine particles in MICC, particularly clay particles, disperse and reconstitute into new particle sizes due to charge interactions and ion exchange processes. These new particle sizes are influenced by factors such as metal ion type, concentration, and surface charge density. This process of dispersion and reconstitution leads to discontinuities in the cumulative volume fraction curve and creates multiple peaks, reflecting significant changes in MICC’s internal structure due to metal ion incorporation.

The influence of different metal ions on MICC’s particle size distribution varies. In this study, zinc ions had the greatest impact on particle size distribution, followed by lead, potassium, and sodium ions. This indicates that different metal ions affect the particle reconstitution process in distinct ways, thereby altering MICC’s particle size distribution characteristics.

#### 3.1.2. SEM

**Figure 5:**
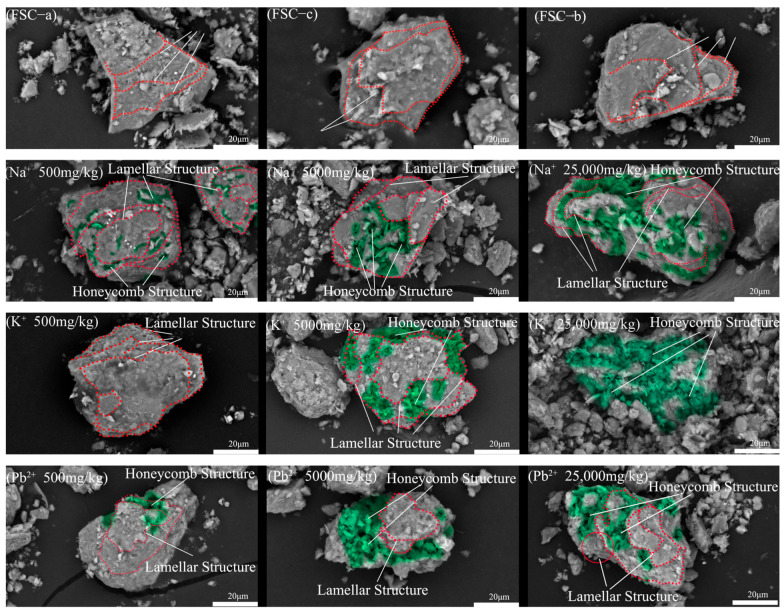

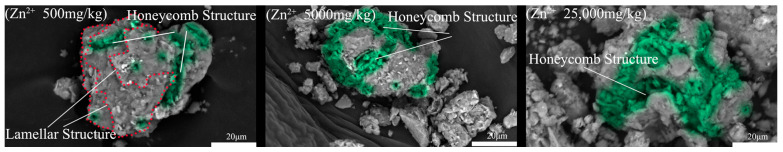
Images of FSC and MICC.

The SEM images (Figure 5) clearly reveal the significant effects of metal ions on the microstructure of soil particles. Red dashed lines indicate the layering of soil particles, while green shading highlights prominent honeycomb structural features. In its natural state, FSC particles exhibit a distinct layered structure, reflecting a stable and ordered particle arrangement. However, once metal ions are introduced, particle interactions change, gradually disrupting the layered structure as metal ion concentrations increase.

This disruption first appears as localized fragmentation of the layered structure and the accumulation of fine particles. Over time, new connections form between particles, leading to the appearance of voids and cracks. As the metal ion concentration rises further, these structural changes become more pronounced, eventually transforming into a layered-honeycomb structure. At high concentrations, the original layered structure nearly disintegrates entirely, forming a typical honeycomb structure.

This structural evolution illustrates the profound impact of metal ions on soil particle interactions. Metal ions interact with the negatively charged particle surfaces, altering the electrical double layer and disrupting the stable layered structure, resulting in fragmentation and reorganization. These changes not only modify the microstructure of MICC but also significantly affect its physical properties.

#### 3.1.3. Specific Surface Area and Mesopore Analysis

Nitrogen adsorption and desorption tests were conducted to determine the microporous structural parameters of FSC and MICC samples, including specific surface area, pore volume, and average pore diameter. These parameters reflect the microporous characteristics of the material, offering key insights into the impact of metal ion incorporation on the structural properties of MICC.

**Figure 6:**
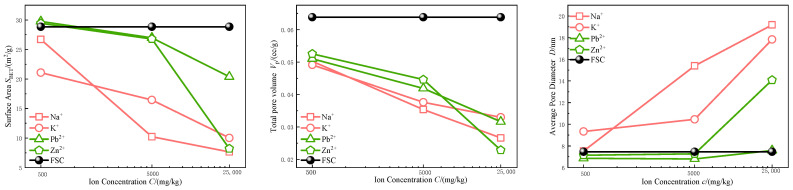
Microporous structural characteristic parameters (specific surface area, pore volume, average pore diameter).

The data from Table 2 and Figure 6 indicate that as the concentration of metal ions increases, the specific surface area and pore volume of the soil significantly decrease, while the average pore diameter tends to increase. Specifically, the specific surface area sharply declines with rising metal ion concentrations. When the concentration reaches 25,000 mg/kg, the MICC (Na^+^) specific surface area reduces by approximately 73.5%; for K^+^ and Zn^2+^, it decreases by 65.3% and 71.6%, respectively. Pb^2+^ has a smaller effect, with only a 29.3% reduction at the same concentration. Additionally, as the concentration of metal ions increases, the total pore volume gradually declines. At 25,000 mg/kg, the total pore volume of Na^+^-contaminated clay decreases by 58.3%, K^+^ by 48.3%, and Pb^2+^ and Zn^2+^ by 50.4% and 64.2%, respectively. Unlike the reductions in specific surface area and pore volume, the introduction of metal ions leads to a significant increase in average pore diameter. At higher concentrations, Na^+^ and K^+^ increase the average pore diameter by 157.3% and 138.9%, respectively, while Zn^2+^ increases it by 88.8%. Pb^2+^, however, shows a more moderate increase.

There are clear differences in how different metal ions affect the clay’s microstructure. Monovalent alkali metal ions like Na^+^ and K^+^ have a more pronounced effect on specific surface area and average pore diameter compared to divalent heavy metal ions like Pb^2+^ and Zn^2+^. Among the heavy metals, Zn^2+^ exerts a much stronger influence on pore structural parameters than Pb^2+^.

This phenomenon can be explained by several mechanisms, including the pore-blocking effect [25], dispersion effect [26], and aggregation effect [17]. Specifically, metal ion deposition can block micropores, leading to their closure and reducing the specific surface area and pore volume. Meanwhile, larger pores may be maintained or expanded, shifting the pore size distribution toward larger dimensions.

Alkali metal ions, due to their lower charge density and weaker adsorption strength, more easily enter the interlayer structure of clay minerals, causing clay particles to expand and increasing the spacing between them. This expansion compresses smaller soil particles, leading to their reorganization and aggregation into larger particles. As a result, the number of small pores decreases, and the originally complex small-pore structure reorganizes into simpler, larger pores.

In contrast, heavy metal ions (e.g., Pb^2+^ and Zn^2+^), with higher charge density and stronger adsorption, compress the double layer and reduce electrostatic repulsion, exhibiting stronger aggregation capabilities. These ions can form bridging structures between soil particles through hydrogen or ionic bonds, promoting particle aggregation into larger structural units. This aggregation increases the number of large pores, further reducing total pore volume and simplifying pore morphology from complex layered or tubular structures.

Although alkali and heavy metal ions act through different mechanisms, both promote the transition of soil micropores from small, complex structures to simpler, larger pores. This change has significant implications for the soil’s physical and mechanical properties, warranting further exploration in future studies.

#### 3.1.4. Cation Exchange Capacity (CEC) Test

In this study, the cation exchange capacity (CEC) of soil particles was measured using the cobalt(III) hexammine chloride extraction–spectrophotometric method. This method involves an ion exchange reaction between cobalt(III) hexammine chloride and the exchangeable cations in the soil. After the reaction, the resulting solution is analyzed via spectrophotometry to accurately quantify the exchangeable cations in the soil.

**Figure 7:**
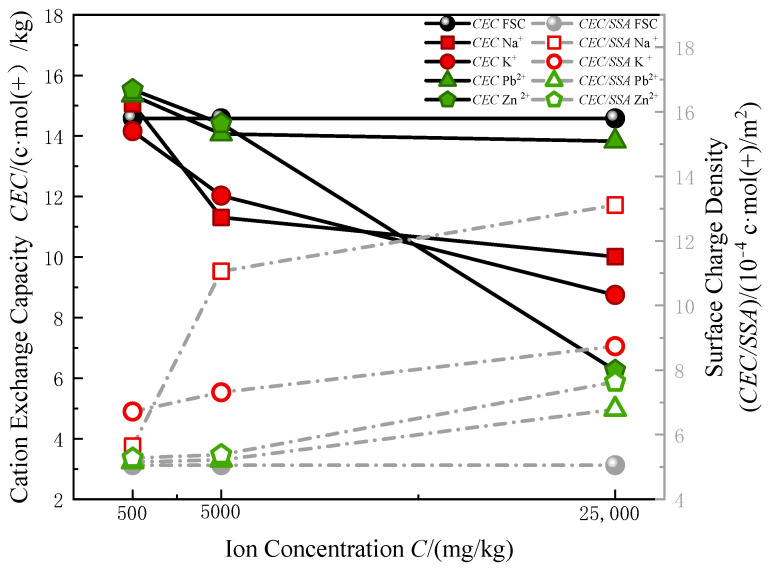
Cation exchange capacity parameters and surface charge density.

After introducing metal ions into the soil, there is a significant reduction in the content of exchangeable cations as the concentration of metal ions increases (Table 3, Figure 7). When the concentration reaches 25,000 mg/kg, the CEC of Zn^2+^-contaminated soil decreases by 57.1%, K^+^-contaminated soil decreases by 39.9%, and Na^+^-contaminated soil decreases by 31.4%. In comparison, the reduction in CEC for Pb^2+^-contaminated soil is relatively smaller, but still shows a noticeable decreasing trend.

The variation in CEC is closely related to the specific surface area of soil particles. A larger specific surface area provides more sites for cation adsorption. As the concentration of metal ions increases, the morphology and pore structure of soil particles change, leading to a reduction in specific surface area and consequently affecting the number of cation adsorption sites. This study evaluated the density of adsorbable cation sites on the soil surface by calculating the ratio of CEC to the specific surface area.

The experimental results indicate that as the concentration of metal ions increases, the density of adsorbable cation sites significantly rises. When the concentration reaches its maximum, the surface charge density of Na^+^-contaminated soil increases by 159.3% and that of K^+^-contaminated soil increases by 72.9%, and the surface adsorption density of Pb^2+^ and Zn^2+^-contaminated soils increases by 34.2% and 50.99%, respectively. In all contaminated samples, this density is higher than that of the uncontaminated field-state clay (FSC) samples.

The surface charge density of MICC containing Na^+^ and K^+^ is significantly higher than that of MICC containing Pb^2+^ and Zn^2+^. This difference arises because heavy metal ions (Pb^2+^, Zn^2+^) carrying more charge have much stronger adsorption forces compared to alkali metal ions (Na^+^, K^+^). Due to this strong adsorption, heavy metal ions tend to become fixed on the surface of soil particles and in the soil pores, limiting their mobility and preventing them from penetrating deeper into the soil structure. In contrast, alkali metal ions, such as Na^+^ and K^+^, shows weaker adsorption, allowing these ions to move more freely and penetrate the interlayer structures of the soil particles. This provides a larger adsorption area for alkali metal ions.

Moreover, the adsorption of heavy metal ions involves not only physical adsorption but also chemical adsorption, which creates a much stronger bond to the soil particle surfaces. This strong binding means heavy metal ions are less likely to be replaced by other ions, reducing their participation in cation exchange. On the other hand, alkali metal ions in MICC are primarily subject to physical adsorption, making them easier to replace through cation exchange.

As a result, due to the larger adsorption area and greater mobility of alkali metal ions, MICC containing Na^+^ and K^+^ exhibits a significantly higher surface charge density than MICC containing Pb^2+^ and Zn^2+^.

This analysis suggests that metal ion contamination not only reduces the CEC of the soil but also significantly alters the surface adsorption charge density, influencing the soil’s microstructure and its capacity to adsorb cations.

### 3.2. Engineering Properties’ Testing

Based on the results of the microstructural tests, further macro-mechanical performance tests were conducted, focusing on compression and shear characteristics. These mechanical performance indicators directly reflect the overall stability and load-bearing capacity of the MICC under different microstructural conditions. By comparing the microstructural and macro-mechanical performance results, a correlation can be established between the micropore morphology and the mechanical behavior of the MICC, revealing the mechanisms by which different metal ions influence the mechanical properties of the MICC.

#### 3.2.1. Void Ratio

Standard consolidation tests were conducted on FSC and four types of MICC under vertical load levels of 50, 100, 200, 400, and 800 kPa to examine the relationship between soil compressibility and factors such as metal ion type, concentration, water content, and dry density under confined conditions.

**Figure 8:**
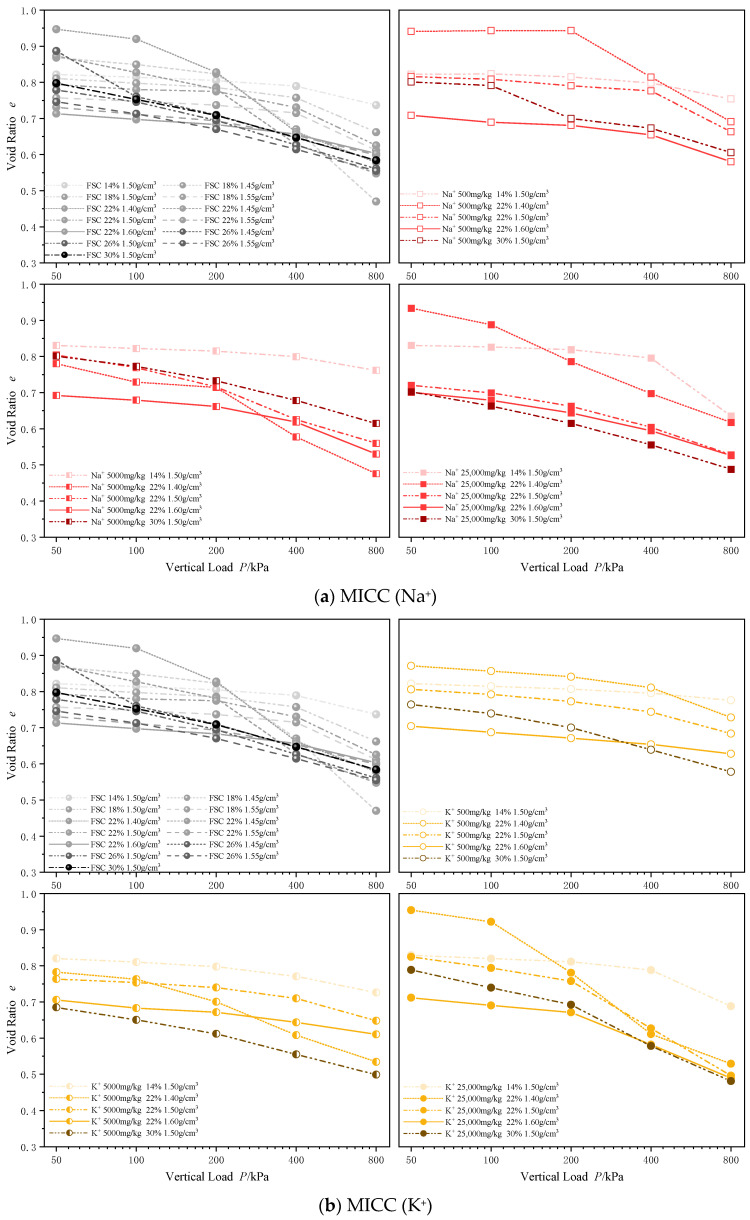

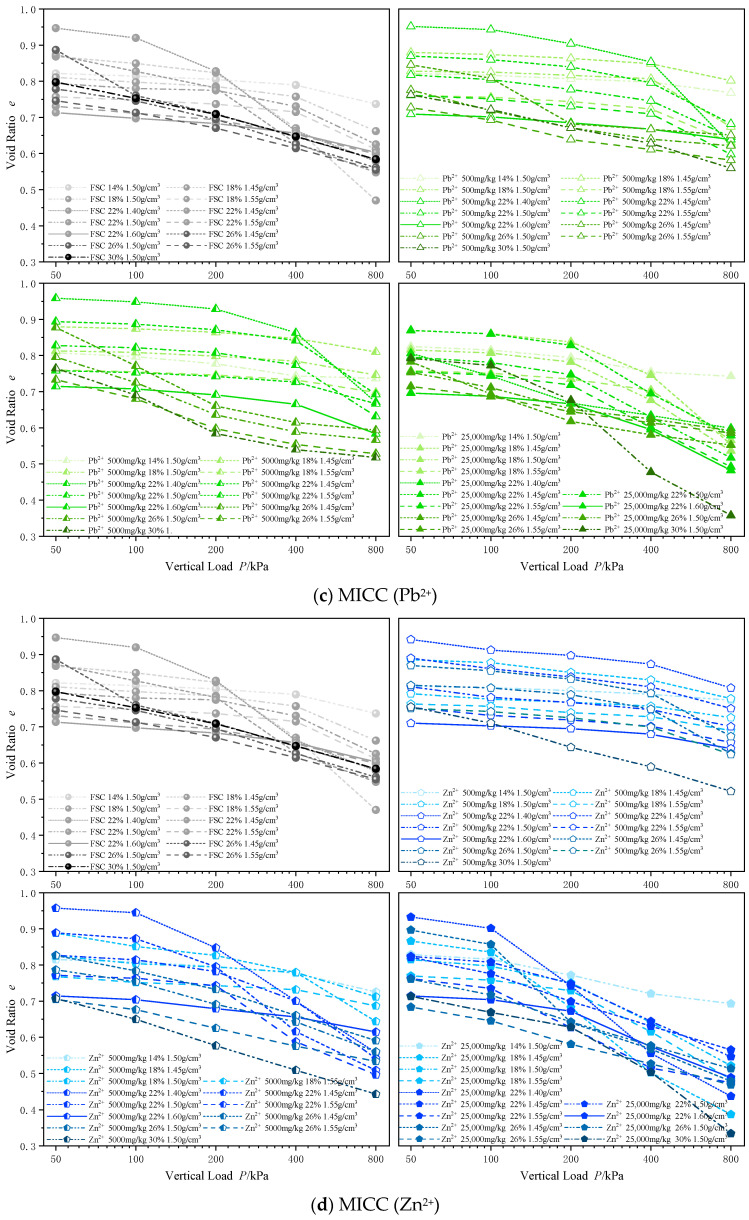
Variation in void ratio during consolidation of MICC.

Figure 8 depicts the changes in void ratio during the consolidation process for FSC and MICC. The figure clearly shows that as the concentration of metal ions increases, the void ratio curve for MICC gradually shifts downward, indicating an increase in soil compressibility. At a metal ion concentration of 500 mg/kg, the void ratio change curve for MICC falls within a range similar to that of FSC. However, when the metal ion concentration reaches 5000 mg/kg, the void ratio changes for Na^+^, K^+^, Zn^2+^, and Pb^2+^ MICC slightly exceed those of FSC under the same load. A further increase in concentration to 25,000 mg/kg causes the MICC void ratio curve to drop significantly below that of FSC, exhibiting maximum compressibility. This is attributed to the higher ion concentration increasing the pore size within the soil, weakening its structural integrity and strength, thereby resulting in a greater change in void ratio. Under an 800 kPa load, the void ratios of Na^+^-, K^+^-, Pb^2+^-, and Zn^2+^-contaminated clays decreased by 16.37%, 18.88%, 38.77%, and 42.69%, respectively. Heavy metal ions, compared to alkali metal ions, have a more pronounced impact on clay compressibility.

As the concentration of metal ions increases, the microstructure of soil particles becomes progressively looser. The pore size within the soil increases, reducing the structural integrity of the soil and leading to a greater change in void ratio. However, alkali metal ions mainly affect soil pores through dispersive action, where many closed micropores remain within the particles, making the pores in alkali-metal-ion-contaminated soils more difficult to compact. In contrast, heavy metal ions influence particle structures through cohesive effects, where intra-aggregate bonding is strong, resulting in high compressive strength. However, the loose inter-aggregate connections make the pores more susceptible to compression, leading to the higher compressibility observed in soils contaminated with heavy metal ions.

As the water content increases and the dry density decreases, the rate of void ratio reduction under external loading accelerates, indicating enhanced soil compressibility. Additionally, a comparison of the void ratio curves under various conditions shows that water content has a more significant impact on void ratio changes than dry density. Furthermore, the introduction of metal ions into the soil tends to concentrate the void ratio change curve, reducing its sensitivity to the water content and dry density during the consolidation process.

#### 3.2.2. Compression Coefficient

When examining the changes in void ratio during the consolidation process of MICC, it is clear that the introduction of metal ions significantly affects the compressibility of the soil. To better understand the relationship between metal ion types, incorporation concentrations, and the compressive properties of the soil, the compression coefficients (*a_v_*) of both FSC and MICC were analyzed across different load levels.

**Figure 9:**
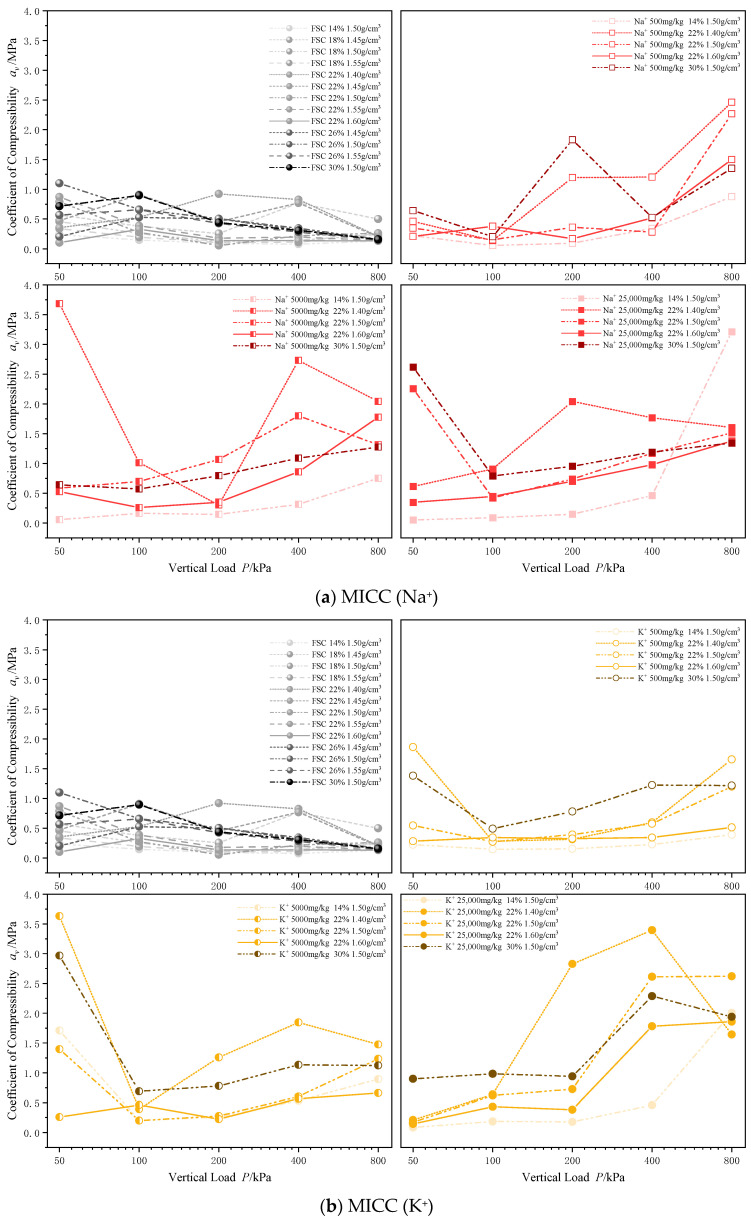

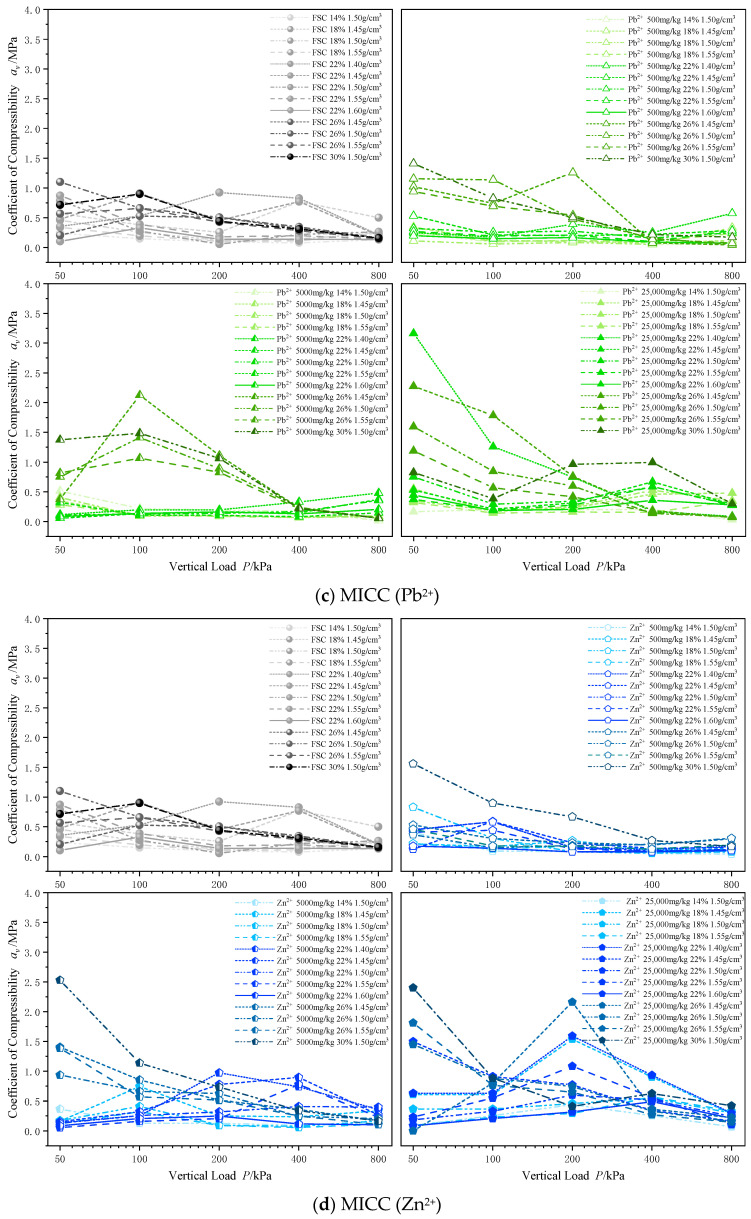
Variation in compression coefficient during consolidation of MICC.

Figure 9 presents the compression coefficient curves for four types of MICC, specifically involving Na^+^, K^+^, Pb^2+^, and Zn^2+^. It is evident that the addition of metal ions significantly increases the soil’s compression coefficient, which continues to rise as the concentration of the metal ions increases. However, there are notable differences between the effects of Na^+^ and K^+^ compared to Pb^2+^ and Zn^2+^ on soil compressibility.

Na^+^ and K^+^, as monovalent alkali metal ions, strongly influence the compression coefficient of the soil across all load levels. As the concentrations of Na^+^ and K^+^ increase, the compression coefficient shows a significant rise under varying load conditions. In contrast, Pb^2+^ and Zn^2+^, being divalent heavy metal ions, primarily impact the compression coefficient under medium to low load conditions. Their introduction results in a significant increase in the compression coefficient at vertical loads of 50 kPa, 100 kPa, and 200 kPa.

This difference can be explained by the dispersive effects of alkali metal ions (Na^+^ and K^+^), which modify the soil structure by weakening the bonding forces between particles. This creates a looser pore structure, with small pores being sealed off and larger pores increasing in number, thereby reducing soil structural integrity and leading to higher compression coefficients across different load levels. In contrast, heavy metal ions (Pb^2+^ and Zn^2+^) primarily alter the soil structure through cohesive effects. These ions form bridging structures between soil particles by creating hydrogen or ionic bonds, which promote the aggregation of particles into larger structural units. Under low load conditions, the strength of these aggregates is greater, leading to pore compression. As the load increases, the pore volume compresses further, resulting in higher strength and lower compression coefficients for the aggregates under high load conditions.

#### 3.2.3. Shear Strength

Standard direct shear tests were conducted on both FSC and four types of MICC under vertical load levels of 50, 100, 200, and 400 kPa. These tests aimed to investigate the relationship between shear strength and factors such as metal ion type, concentration, moisture content, and dry density in the soil samples.

**Figure 10:**
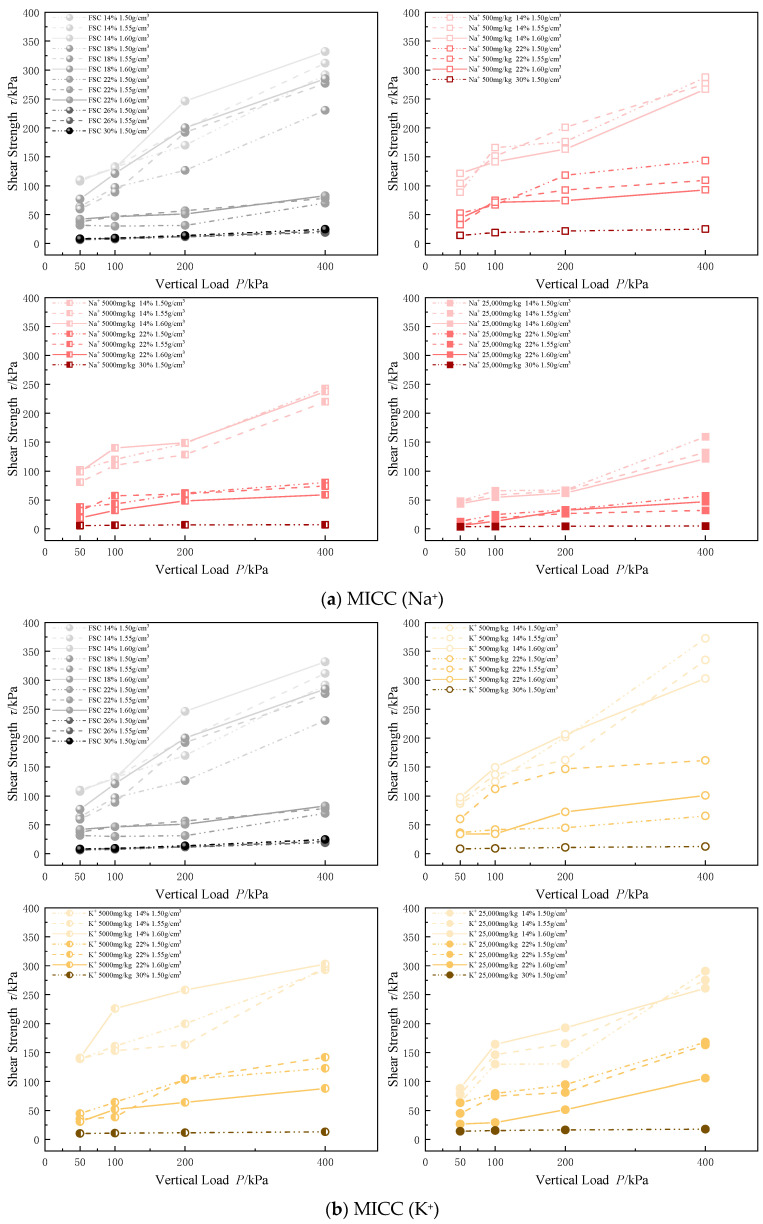

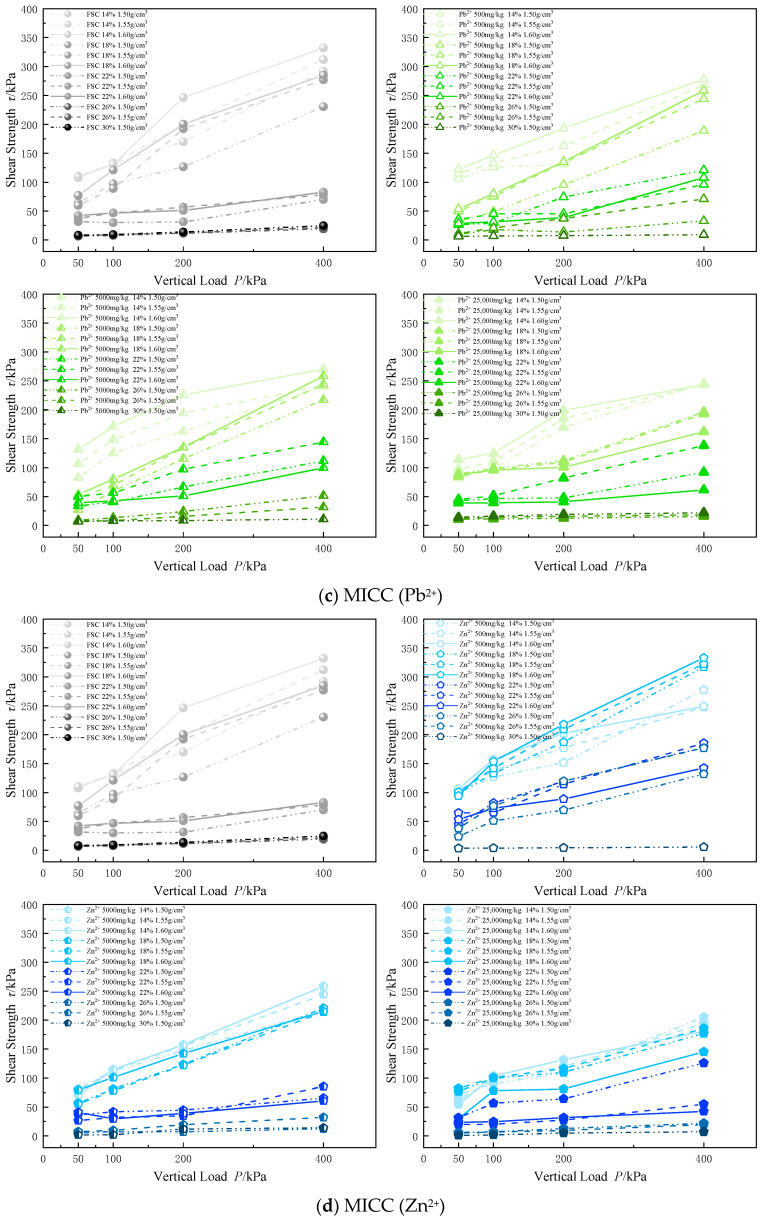
Variation in shear strength of MICC.

Based on the analysis of Figure 10, it is evident that the incorporation of metal ions significantly reduces the shear strength of the clay, with the effect intensifying as the concentration of metal ions increases. The impact of different metal ions on shear strength varies, with the observed order in this experiment being Na^+^ > Zn^2+^ > Pb^2+^ > K^+^. Under a 400 kPa load, the shear strength of MICC decreases by a maximum of 79.11% (Na^+^), 71.08% (Zn^2+^), 31.22% (Pb^2+^), and 28.91% (K^+^).

The introduction of metal ions alters the surface characteristics of the particles, weakening the microstructure of the soil. This results in a looser internal particle structure and reduced interparticle bonding strength, which, at the macro level, leads to a decrease in the overall shear strength of the soil. The varying effects of different metal ions on soil shear strength are attributed to several factors, including the composition of soil particles, surface functional groups, and initial structure, as well as the ion’s charge density, ionic radius, and hydration capacity.

From the distribution and dispersion of the shear strength curves, it can be observed that under the same loading conditions, clay with a lower moisture content and higher dry density typically exhibits a greater shear strength, internal friction angle, and cohesion. Furthermore, shear strength is more sensitive to changes in moisture content than to changes in dry density.

#### 3.2.4. Cohesion and Internal Friction Angle

The incorporation of metal ions has a significant negative impact on the shear strength of soil. To reveal the specific mechanisms by which different types and concentrations of ions affect shear strength, this section focuses on analyzing the trends and correlations between metal ion concentration and type, and the cohesion and internal friction angle of the soil.

Water content and dry density are key factors influencing the internal friction angle and cohesion of soils. Typically, soils with a lower water content and higher dry density exhibit higher internal friction angles and cohesion. This is because a lower water content implies a thinner layer of adsorbed water on particle surfaces and less free water between particles, increasing direct particle contact and enhancing friction. And a higher dry density indicates a tighter soil structure with reduced particle spacing, strengthening mechanical interlocking and interactions among soil particles.

**Figure 11:**
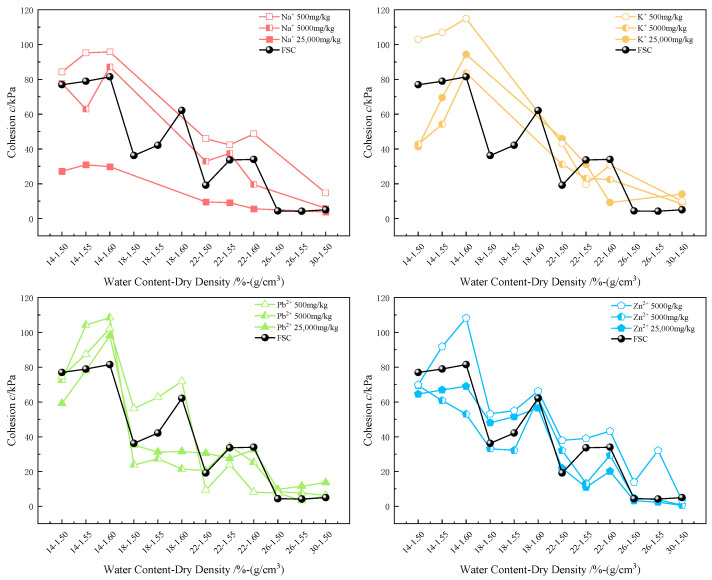
Variation in cohesion of MICC.

**Figure 12:**
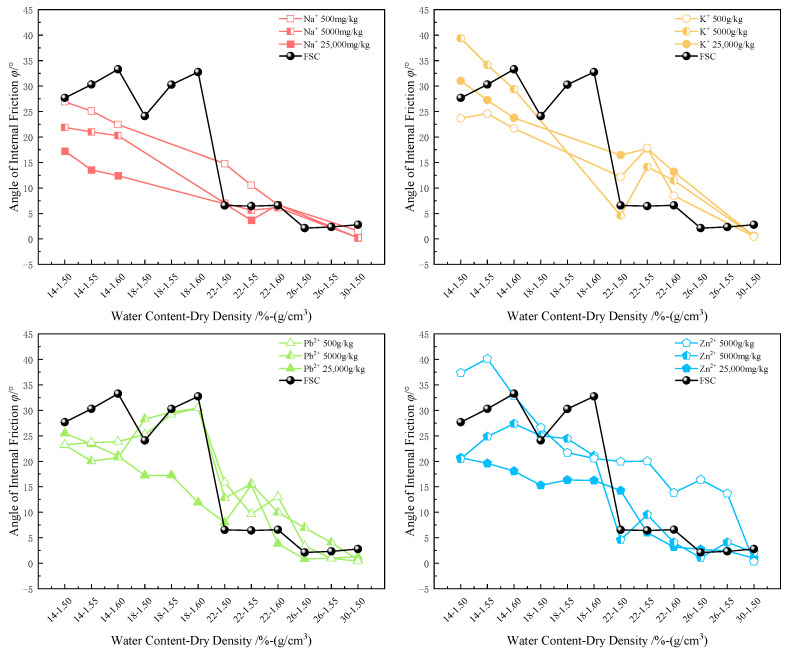
Variation in internal friction angle of MICC.

According to the data analysis from Figure 11 and Figure 12, increasing metal ion concentrations lead to significant changes in the cohesion and internal friction angle of the soil. Specifically, as the concentration of metal ions in the soil rises, both the cohesion and internal friction angle decrease notably. However, at lower concentrations, soil cohesion shows a slight increase, slightly higher than in the FSC.

At low concentrations, alkali metal ions (e.g., Na^+^, K^+^) form hydration layers that increase particle spacing, slightly reducing electrostatic repulsion between soil particles. In contrast, heavy metal ions (e.g., Pb^2+^, Zn^2+^) enhance attractive forces between particles through bridging effects. These combined mechanisms result in a moderate increase in the cohesion of MICC at low concentrations. However, as metal ion concentrations further increase, alkali metal ions cause soil particles to swell and disperse, resulting in a looser and more unstable structure. Meanwhile, heavy metal ions promote tighter bonding between particles through coagulation, forming larger aggregates, but the connections between these aggregates remain relatively weak. Consequently, both effects lead to a decrease in overall cohesion at medium to high concentrations.

In contrast, the effect of metal ions on the internal friction angle is more distinct. As metal ion concentrations rise, the internal friction angle of the soil shows a clear downward trend. Under low moisture conditions (14–18%), the internal friction angle of MICC is generally lower than that of FSC, while under high moisture conditions (22–30%), the internal friction angle of MICC is typically higher than that of FSC. With increasing metal ion concentrations, both the dispersive effects of alkali metal ions and the coagulative effects of heavy metal ions become more pronounced, loosening particle bonds and reducing frictional resistance between larger aggregates, leading to a decline in the internal friction angle.

Under low moisture conditions, metal ions increase the thickness of the adsorbed water layer between particles, reducing direct particle contact and lowering frictional resistance. Conversely, under high moisture conditions, metal ions enhance the formation of a stronger adsorbed water layer on soil particles. The incompressible water supports the particle structure, increasing frictional resistance, thereby raising the internal friction angle compared to uncontaminated soil.

The incorporation of metal ions notably alters the microstructure, often leading to a looser and less stable soil structure, negatively affecting its engineering properties. This research not only advances our understanding of soil behavior under different conditions but also provides a theoretical basis for the more accurate prediction and control of soil performance in practical engineering applications.

## 4. Analysis of Hydro-Pore Structural Parameter and Micropore Morphology Index

The theory of pore structure suggests that the macro strength of soil is primarily determined by its pore structure. However, existing evaluation parameters often fail to fully integrate both microscopic pore morphology and macroscopic pore states. Experimental results indicate that the type and concentration of inorganic metal ions have a significant impact on the microscopic pore structure of the soil. These ions reduce the specific surface area and pore volume while increasing the average pore size. Changes in the microscopic pore structure are often accompanied by alterations in the inter-particle bonding strength, profoundly affecting the micro-mechanical properties of the soil. To better describe these changes in the soil’s microstructure, the Micropore Morphology Index (MMI) $I_{mmi}$ is proposed:

(1) $$I_{mmi}=\ln\frac{A_{S}\cdot D}{a\cdot V_{P}}$$

where $I_{mmi}$ is the Micropore Morphology Index (dimensionless); $A_{S}$ is the specific surface area of the particles (m^2^/g); D is the average pore diameter (nm); $V_{P}$ is the pore volume (cc/g); and is an adjustment coefficient (a = 1000).

MMI reflects the relative distribution of particle surface area within a unit pore volume, providing a comprehensive description of the complexity and geometry of the soil’s pore structure. There is a dynamic balance between specific surface area, pore volume, and average pore size. As the concentration of metal ions increases, soil particles tend to aggregate or rearrange, leading to a gradual reduction in both specific surface area and pore volume, while the average pore size increases. Consequently, the MMI rises. This process indicates that smaller pores gradually merge, causing the pore structure to become coarser, with larger pores dominating and the overall pore structure becoming more heterogeneous.

At the macro scale, the void ratio and degree of saturation are key parameters influencing the mechanical strength of soil. An increase in void ratio typically indicates a larger internal pore volume and fewer contact points between particles, which weakens the overall strength of the soil. The degree of saturation reflects the extent to which the pores are filled with water. As saturation increases, pore water pressure rises, reducing the effective stress and friction between particles, thereby affecting the strength of the soil. Based on pore structure theory and combining the microscopic pore structure with macroscopic pore states, the hydro-pore structural parameter (HPSP) $P_{hp}$ is proposed:

(2) $$P_{hp}=b\cdot S_{r}\cdot e^{I_{mmi}}=b\cdot S_{r}\cdot e^{\ln_{e}\frac{A_{s}\cdot D}{a\cdot V_{p}}}$$

where $P_{hp}$ is the hydro-pore structural parameter (dimensionless); $S_{r}$ is the degree of saturation of the soil; e is the void ratio; $I_{mmi}$ is the Micropore Morphology Index; and b is an adjustment coefficient (b = 10).

HPSP comprehensively describes the soil’s microscopic pore structure, macroscopic pore state, and water content characteristics. It effectively reflects the coupled interaction between the water content and microscopic pore morphology, while sensitively revealing the impact of microscopic structural changes on the macro-mechanical properties of the soil.

**Figure 13:**
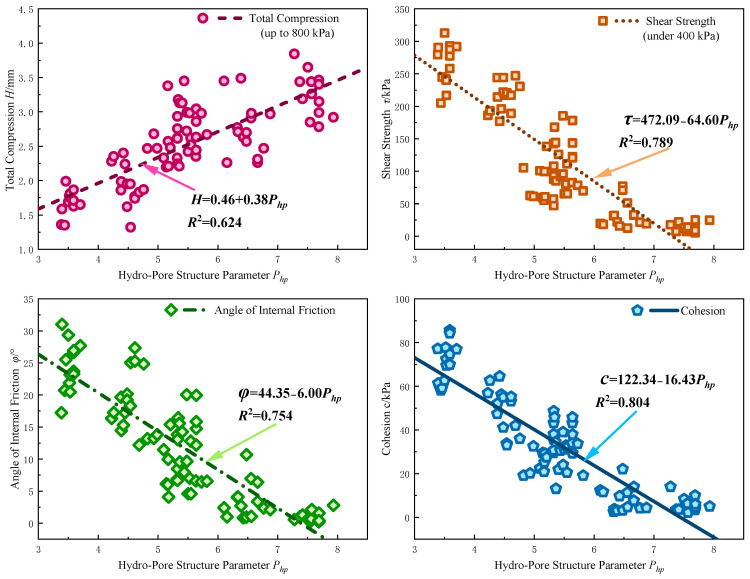
Relationship between hydro-pore structural parameter and mechanical parameters.

As shown in Figure 13, with the increase in HPSP, the compression of the soil (under an 800 kPa load) increases significantly, while the shear strength (under a 400 kPa load), internal friction angle, and cohesion gradually decrease. This trend indicates that HPSP not only effectively reflects the soil’s microscopic pore structure and water content characteristics but also accurately assesses changes in the soil’s macro-mechanical properties under different conditions.

Firstly, the increase in HPSP leads to a significant linear rise in the compression, which is closely related to changes in the soil pore structure.

(3) $$H=0.46+0.38P_{hp}$$

where H is the total compression under an 800 kPa uniaxial load (mm); and $P_{hp}$ is the hydro-pore structural parameter. The correlation coefficient *R*^2^ = 0.624.

As larger pores gradually dominate the internal structure of the soil, the contact area between particles decreases, and the arrangement of particles becomes looser, weakening the mutual support between them. This structural change makes the soil more prone to deformation under load, resulting in an increase in compression. Additionally, as the degree of saturation increases, the pore water pressure rises, causing a substantial reduction in effective stress between particles, further weakening their load-bearing capacity, which exacerbates the compressibility of the soil.

Secondly, the increase in HPSP causes a significant linear decrease in shear strength:

(4) $$\tau =472.09-64.60P_{hp}$$

where τ is the shear strength of the soil under a 400 kPa uniaxial load (kPa). The correlation coefficient *R*^2^ = 0.789.

The primary reason for the reduction in shear strength is the loosening of the microscopic pore structure and the lubricating effect of water. The redistribution of microscopic pores, along with the increase in larger pores, reduces the contact points and interaction forces between particles, weakening the soil’s ability to resist failure under shear stress. Additionally, the water filling the pores acts as a lubricant, and particularly in highly saturated conditions, the increase in pore water pressure further lowers the friction and effective stress between particles, making the soil more prone to shear failure.

Finally, regarding the internal friction angle and cohesion, the increase in HPSP also exhibits a significant weakening effect:

(5) $$\psi =44.35-6.00P_{hp}$$

(6) $$c=122.34-16.34P_{hp}$$

where ψ is the internal friction angle (°), and c is the cohesion (kPa). The correlation coefficient for the internal friction angle is *R*^2^ = 0.754, and for cohesion, it is *R*^2^ = 0.804.

The reduction in the internal friction angle indicates a weakening of the frictional forces between particles, which is caused by the loosening of the pore structure and a decrease in inter-particle bonding strength. Additionally, the formation of a water film on the particle surfaces reduces frictional resistance between particles, further diminishing the soil’s shear strength. The decrease in cohesion reflects a significant reduction in the bonding forces between particles. As the HPSP value increases, the effective contact area between particles decreases, weakening the adhesive and attractive forces between them. At the same time, the increase in saturation exacerbates this process. The thickening of the bound water layer reduces electrostatic attraction and chemical bonding forces, further lowering the bonding strength between particles and significantly reducing the cohesion of the soil.

In summary, there is a clear linear relationship between HPSP and the soil’s compression amount, shear strength, internal friction angle, and cohesion. The increase in HPSP is accompanied by the redistribution of the soil’s pore structure and changes in moisture conditions, leading to significant alterations in the soil’s mechanical properties. Specifically, this is manifested by increased compressibility, reduced shear strength, and decreases in both internal friction angle and cohesion. As a comprehensive evaluation parameter, HPSP effectively reflects the mechanical behavior of the soil under different pore and moisture conditions, providing important theoretical support for the study of soil strength and stability.

## 5. Conclusions

The introduction of sodium (Na^+^), potassium (K^+^), lead (Pb^2+^), and zinc (Zn^2+^) ions causes significant changes in the microstructure of clay, leading to a reduction in particle size and an increase in clay content. The clay structure transitions from a layered arrangement to a dispersed, honeycomb-like configuration. Additionally, the number of small pores decreases while larger pores dominate. At a metal ion concentration of 25,000 mg/kg, the MICC’s (Na^+^, K^+^, Pb^2+^, and Zn^2+^) specific surface area reduces by 29.3–73.5%, the total pore volume shrinks by 48.3–58.3%, and the average pore diameter increases by 88.8–157.3%.

The cation exchange capacity also decreases as specific surface area declines. However, surface charge density, indicating the number of adsorbable cations per unit of surface area, increases significantly with rising metal ion concentrations, showing gains of 34.2–159.3%.

The effects of different metal ions on clay’s microstructure vary. Alkali metal ions (Na^+^ and K^+^) primarily induce dispersion, while heavy metal ions (Pb^2+^ and Zn^2+^) lead to particle coagulation. This difference in mechanism influences the void ratio and compressibility of the clay. Alkali metal ions, with their dispersive effect, result in higher compression coefficients across all load levels, whereas heavy metal ions form larger aggregates, which are more compressible under low loads but show reduced compressibility under higher loads.

In terms of mechanical properties, the shear strength of MICC (Na^+^, K^+^, Pb^2+^, and Zn^2+^) decreases by 28.91–79.11% compared to FSC. Both shear strength and the internal friction angle decrease with an increasing metal ion concentration. Although cohesion may be slightly higher than that of FSC at lower concentrations, it decreases significantly as the ion concentration rises.

The Micropore Morphology Index (MMI) was introduced to quantify the complexity and geometry of the pore structure in MICC. MMI increases with the addition of metal ions and rises further as the concentration of metal ions increases. An increase in MMI suggests that larger pores dominate the structure, with a tendency toward simplification.

The hydro-pore structural parameter (HPSP), which integrates the MMI, void ratio, and degree of saturation, offers a comprehensive representation of the soil’s microscopic structure, pore state, and water content. This parameter demonstrates a clear linear relationship with key mechanical properties such as compression amount, shear strength, internal friction angle, and cohesion.

## Figures and Tables

**Table 1:** Metal ion configuration table.

Metal Ion Type	Metal Ion Concentration C/(mg/kg)	Compound	Molecular Weight *M*_r_
Na^+^	500 mg/kg 5000 mg/kg 25,000 mg/kg	NaCl	22.99/58.44
K^+^		KCl	39.10/74.55
Pb^2+^		Pb(NO_3_)_2_	207.2/331.22
Zn^2+^		Zn(NO_3_)_2_·6(H_2_O)	65.38/297.52

**Table 2:** Microporous structural characteristic parameters.

	Ion Concentration *C*/(mg/kg)	Specific Surface Area *A*s/(m^2^/g)	Total Pore Volume *V*_p_/(cc/g)	Average Pore Diameter *D*/nm
FSC	0	28.837	6.381 × 10^−2^	7.46268
Na^+^	500	26.703	5.022 × 10^−2^	7.52264
	5000	10.224	3.536 × 10^−2^	15.4001
	25,000	7.635	2.6623 × 10^−2^	19.1890
K^+^	500	21.082	4.912 × 10^−2^	9.32054
	5000	16.453	3.762 × 10^−2^	10.4546
	25,000	10.009	3.300 × 10^−2^	17.8319
Pb^2+^	500	29.754	5.101 × 10^−2^	6.85744
	5000	26.977	4.188 × 10^−2^	6.80223
	25,000	20.383	3.162 × 10^−2^	7.57922
Zn^2+^	500	29.421	5.251 × 10^−2^	7.13979
	5000	26.768	4.460 × 10^−2^	7.26219
	25,000	8.200	2.288 × 10^−2^	14.0897

**Table 3:** Cation exchange capacity parameters and surface charge density.

	Ion Concentration C/(mg/kg)	Specific Surface Area (SSA) As/(m^2^/g)	Cation Exchange Capacity (CEC) c·mol(+)/kg	Surface Charge Density (CEC/SSA) 10^−4^ c·mol(+)/m^2^
FSC	0	28.837	14.58	5.056
Na^+^	500	26.703	15.07	5.643
	5000	10.224	11.31	11.062
	25,000	7.635	10.01	13.111
K^+^	500	21.082	14.16	6.717
	5000	16.453	12.03	7.312
	25,000	10.009	8.75	8.742
Pb^2+^	500	29.754	15.35	5.159
	5000	26.977	14.07	5.216
	25,000	20.383	13.83	6.785
Zn^2+^	500	29.421	15.53	5.278
	5000	26.768	14.41	5.383
	25,000	8.200	6.26	7.634

## Data Availability

The original contributions presented in the study are included in the article, further inquiries can be directed to the corresponding author.

## References

[1] Carnier R., de Abreu C.A., de Andrade C.A., Fernandes A.O., Silveira A.P.D., Coscione A.R. (2023). Soil quality index as a tool to assess biochars soil quality improvement in a heavy metal-contaminated soil. Environ. Geochem. Health.

[2] Proshad R., Li J., Sun G. (2024). Field application of hydroxyapatite and humic acid for remediation of metal-contaminated alkaline soil. Environ. Sci. Pollut. Res..

[3] Emenike C.U., Agamuthu P., Fauziah S.H., Omo-Okoro P.N., Jayanthi B. (2023). Enhanced Bioremediation of Metal-Contaminated Soil by Consortia of Proteobacteria. Water Air Soil Pollut..

[4] Taharia, Dey D., Das K., Sukul U., Chen J.-S., Banerjee P., Dey G., Sharma R.K., Lin P.-Y., Chen C.-Y. (2024). Microbial induced carbonate precipitation for remediation of heavy metals, ions and radioactive elements: A comprehensive exploration of prospective applications in water and soil treatment. Ecotoxicol. Environ. Saf..

[5] Chen Y., Zuo M., Yang D., He Y., Wang H., Liu X., Zhao M., Xu L., Ji J., Liu Y. (2024). Synergistically Effect of Heavy Metal Resistant Bacteria and Plants on Remediation of Soil Heavy Metal Pollution. Water Air Soil Pollut..

[6] Wang Y., Li A., Ren B., Han Z., Lin J., Zhang Q., Cao T., Cui C. (2022). Mechanistic insights into soil heavy metals desorption by biodegradable polyelectrolyte under electric field. Environ. Pollut..

[7] Yarlagadda P.S., Matsumoto M.R., Vanbenschoten J.E., Kathuria A. (1995). Characteristics of Heavy Metals in Contaminated Soils. J. Environ. Eng..

[8] Wu Y., Pan Z., Zhu C., Ding S., Zhang Z., Hu K. (2020). Research Progress on Engineering Properties of Heavy Metal Contaminated Soil. J. Henan Univ. (Nat. Sci.).

[9] Han L., Zhang W., Wang X., Yang X., Yuan Y., Xie D. (2023). Response of physical and mechanical parameters of saline soil to the salt content variation and its microstructure characteristics. J. Qinghai Univ..

[10] Zha F., Zhu F., Xu L. (2021). Laboratory study of strength, leaching, and electrical resistivity characteristics of heavy-metal contaminated soil. Environ. Earth Sci..

[11] Ma Q., Wu N., Xiao H., Li W., Xiang J. (2022). Effect of Zn^2+^-Cu^2+^ combined heavy metal on mechanical properties and microstructure of clayey soil. J. Mt. Sci..

[12] Zhang D., Xiao G., Wu Y., Xu G., Liu W. (2023). Compression deformation mechanisms of red clay driven by heavy metal Cu^2+^. Rock Soil Mech..

[13] Feng C., Li J., Liu J., Xue Q. (2022). Experimental study on the compaction characteristics and microstructure of arsenic and cadmium co-contaminated soil. Rock Soil Mech..

[14] Chu Y., Liu S., Wang F., Cai G., Bian H. (2017). Estimation of heavy metal-contaminated soils’ mechanical characteristics using electrical resistivity. Environ. Sci. Pollut. Res..

[15] Ma Q., Hu X., Xing W. (2017). Experimental Study on Engineering Mechanical Properties of Different Concentrations of Zinc Ion Contaminated Soil. Saf. Environ. Eng..

[16] Shen J., Wan B., Liu S., Wang Z., Fu J. (2021). Rock and Soil Mechanics. Rock Soil Mech..

[17] Li J., Xue Q., Wang P., Li Z. (2015). Effect of lead (II) on the mechanical behavior and microstructure development of a Chinese clay. Appl. Clay Sci..

[18] Zha F., Yang Z., Kang B., Shen Y. (2024). Electrical resistivity evaluation of MICP solidified lead contaminated soil. Environ. Earth Sci..

[19] Li J., Kang B., Zha F. (2024). Strength and leaching characteristics of micp-solidified lead-contaminated soils under the action of freeze-thaw cycles. J. Eng. Geol..

[20] (2018). Soil Environmental Quality Risk Control Standard for Soil Contamination of Development Land.

[21] (2017). Soil Quality—Determination of Cation Exchange Capacity (CEC)—Hexamminecobalt Trichloride Solution-Spectrophotometric Method.

[22] (2020). Standard Test Methods for One-Dimensional Consolidation Properties of Soils Using Incremental Loading.

[23] (2019). Standard for Geotechnical Testing Method.

[24] (2023). Standard Test Method for Direct Shear Test of Soils Under Consolidated Drained Conditions.

[25] Alizadeh A.H., Akbarabadi M., Barsotti E., Piri M., Fishman N., Nagarajan N. (2018). Salt Precipitation in Ultratight Porous Media and Its Impact on Pore Connectivity and Hydraulic Conductivity. Water Resour. Res..

[26] Parameswaran T.G., Sivapullaiah P.V. (2017). Influence of Sodium and Lithium Monovalent Cations on Dispersivity of Clay Soil. J. Mater. Civ. Eng..

